# Neurosurgical Management and Outcome Parameters in 237 Patients with Spondylodiscitis

**DOI:** 10.3390/brainsci11081019

**Published:** 2021-07-30

**Authors:** Mirza Pojskić, Barbara Carl, Vincent Schmöckel, Benjamin Völlger, Christopher Nimsky, Benjamin Saβ

**Affiliations:** 1 Department of Neurosurgery, University of Marburg, 65199 Marburg, Germany; Barbara.carl@helios-gesundheit.de (B.C.); vincentschmoeckel@hotmail.com (V.S.); voellger@med.uni-marburg.de (B.V.); nimsky@med.uni-marburg.de (C.N.); sassb@med.uni-marburg.de (B.S.); 2 Marburg Center for Mind, Brain and Behavior (MCMBB), 65199 Marburg, Germany; 3 Department of Neurosurgery, Helios Dr. Horst Schmidt Kliniken, 65199 Wiesbaden, Germany

**Keywords:** spondylodiscitis, spinal empyema, antibiotic therapy, osteomyelitis, infection, spine, operative therapy

## Abstract

Surgical treatment of spondylodiscitis allows for rapid mobilization and shortens hospital stays, which makes surgical treatment the first-line therapy. We aim to describe our experiences with operative treatment on spondylodiscitis and to determine the parameters that are important in the prediction of outcomes. A retrospective review identified 237 patients who were operatively treated for spondylodiscitis in our institution between January 2010 and December 2018. Clinical data were collected through review of electronic records and relevant imaging. In all cases, contrast-enhancing MRI from the infected region of the spine was obtained. Leukocyte count and C-reactive protein concentrations (CRP) were determined in all the patients. We included 237 patients in the study, 87 female (36.7%) and 150 male (63.3%), with a mean age of 71.4 years. Mean follow-up was 31.6 months. Forty-five patients had spondylodiscitis of the cervical, 73 of the thoracic, and 119 of the lumbosacral spine. All the patients with spondylodiscitis of the cervical spine received instrumentation. In thoracic and lumbar spine decompression, surgery without instrumentation was performed in 26 patients as immediate surgery and in a further 28 patients in the early stages following admission, while 138 patients received instrumentation. Eighty-nine patients (37.6%) had concomitant infections. Infection healing occurred in 89% of patients. Favorable outcomes were noted in patients without concomitant infections, with a normalized CRP value and in patients who received antibiotic therapy for more than six weeks (*p* < 0.05). Unfavorable outcomes were noted in patients with high CRP, postoperative spondylodiscitis, and recurrent spondylodiscitis (*p* < 0.05). Application of antibiotic therapy for more than six weeks and normalized CRP showed a correlation with favorable outcomes, whereas concomitant infections showed a correlation with unfavorable outcomes. A detailed screening for concomitant infectious diseases is recommended.

## 1. Introduction

Spondylodiscitis is a potentially life-threatening infection that has high morbidity rates [1]. While vertebral osteomyelitis is rare, at a rate of 3–5%, it is the third most common form of osteomyelitis at >50 years of age [2,3]. Spondylodiscitis is usually a monobacterial infection and more than 50% of cases in Europe are caused by Staphylococcus aureus, followed by Gram-negative pathogens such as Escherichia coli (11–25%) [4,5]. The most common pathogen worldwide is *Mycobacterium tuberculosis* [4]. Brucellosis is endemic in the Mediterranean [6]. Rare causes include fungal infections like Coccidioidomycosis [7] and secondary syphilis [8]. While the literature has reported a low incidence of approximately 5–6/100,000 patient years, recent data are clearly higher at an age-standardized rate of approximately 30/250,000 [9]. The rate of surgical procedures in older, polymorbid patients is rising, and patients aged over 65 years are affected up to 3.5 times more frequently, while women are affected 0.82 times less frequently [10]. Other risk factors include diabetes mellitus, immunosuppression, a history of infections, intravenous drug abuse, and HIV infection [9]. Lethal outcomes occur almost exclusively in the older age group [11].

Spontaneous and iatrogenic spondylodiscitis are becoming more frequent, yet there are no definite treatment guidelines [12,13]. Diagnosis is based on history and physical examinations, laboratory data, proper imaging, and culture. In the past, most infections were treated with an appropriate course of antibiotics and bracing if needed. An operative treatment of spondylodiscitis may be favorable in certain conditions, but it is not undisputedly to be performed in each case [9,14]. Neurologic deficits, sepsis, intraspinal empyema, the failure of conservative treatment, and spinal instability are all indications that surgical treatment is suitable [9]. While surgical treatment of spondylodiscitis has its advantages, conservative therapy with immobilization, bracing, and intravenous antibiotic therapy, followed by a course of oral antibiotic therapy for six weeks, is still considered to be an effective therapy [14], at least for patients with mild to moderate symptoms, with an absence of spinal instability and empyema or sepsis, as well as for patients with monosegmental infection. An image-guided or intraoperative aspiration or biopsy of a disc space or vertebral endplate sample often establishes the microbiological and pathological diagnosis of native vertebral osteomyelitis [15]. Surgical treatment of spondylodiscitis allows for rapid mobilization and shortened hospital stays [16], which leads to a paradigm change in the treatment and has made surgical treatment the first-line therapy. Complete healing of the infection with a normalization of the laboratory infection parameters, and with the application of antibiotic therapy for a minimum of six weeks, is the second pillar in the treatment of spondylodiscitis [17]. Screening for associated infectious diseases in patients with spondylodiscitis has been recently advocated [18]. With increasing experience in spinal instrumentation and fusion techniques, surgical management needs to be revisited [19]. This retrospective study aims to assess the effectiveness of surgical treatment, as well as to identify prognostic parameters, which correlate with mid and long-term outcomes.

## 2. Materials and Methods

A retrospective review of patients who underwent surgical treatment in our department in the period between January 2010 and December 2018 was performed. Two hundred and thirty-seven consecutive cases were identified, and data were collected through review of patient’s electronic records and relevant imaging. In all the cases, Gadolinium contrast-enhancing MRI from the infected region of the spine was obtained. Leukocyte count and C-reactive protein concentrations (CRP) were determined in all patients using routine laboratory techniques. The leukocyte count and CRPs were standardly measured prior to surgery, as well as every 2–3 days following surgery, in order to discharge. These parameters were also obtained up to the follow-up in ambulatory setting for infection control assessment. A normal leukocyte count was defined as <10 G/L and a normal CRP value was defined as <5 mg/L. For the final assessment, CRP values prior to surgery, at the time of the switch from intravenous to oral antibiotic therapy, as well as at the last follow-up, were taken into consideration for statistical analysis.

Indications for surgery included the presence of pain, neurological deficits, sepsis, radiological signs of compression of the spinal cord, spinal instability, and the presence of empyema. In cases of instability, instrumentation surgery was performed. Furthermore, in cases without instability of the spine, stabilization surgery for segmental immobilization was performed as an analogue to immobilization with a corset in non-surgical treatment. CT scans were regularly obtained for all the patients who underwent spinal stabilization with screws, rods, and cages following surgery. Conservative treatment with immobilization was advised to patients as an alternative therapy option. Application of antibiotic therapy was performed in coordination with microbiologists. Broad-spectrum antibiotic therapy, which covers Gram-positive (Vancomycin), multiresistant Gram-positive (Linezolid), Gram-negative (Meropenem), and multiresistant Gram-negative pathogens (Fosfomycin) with solid bone and liquor accessibility, was initially used. Antibiotic therapy was initially performed intravenously (IV) as a broad-spectrum therapy (standard use of Vancomycin 3 × 1 g IV or Linezolid 2 × 600 mg IV, +Meropenem 3 × 1 g i.v + Fosfomycin 3 × 5 g IV), followed by targeted therapy tailored according to the antibiogram in cases where pathogens could be isolated. Antibiotic therapy was switched to oral administration (oral antibiotics tailored to antibiogram or broad spectrum antibiotics in cases where the pathogen was not isolated) after clear improvement to the clinical and laboratory parameters and applied until clinical improvement and significant regression of infection parameters occurred. Screening for further foci was performed using contrast-enhancing computer tomography (CT) of the thorax and abdomen, trans-esophageal echocardiography (TEE), urinary status, as well as a clinical examination of the nasal cavities, dental status, and craniofacial sinuses. Complete regression of the infection was defined as a significant decline in the infection parameters (leukocyte count and CRP concentration) without clinical and/or radiological signs of infection at the follow-up and at a minimum of four months after surgery. In addition, the neurological status was determined postoperatively at a minimum of four months following surgery. The neurological status of patients with spine diseases at our clinic is standardized and contains an examination of motoric deficits, which are defined into grades of muscle strength 1–5 for muscles of the upper and lower extremity (0 = complete paralysis, 1 = flicker of contraction, 2 = contraction with gravity eliminated alone, 3 = contraction against gravity alone, 4 = contraction against gravity and some resistance, and 5 = contraction against powerful resistance for deltoid, biceps, and triceps muscle, iliopsoas, quadriceps, as well as foot dorsiflexion, plantar flexion, eversion and inversion and toe plantar flexion and dorsiflexion. Furthermore, examination of the spinal ataxia in patients who were able to walk (tightrope and blind walk), examination of the coordination of upper and lower extremities using a finger nose test and knee heel try, the sensory exam with a comparing of tactile sensations between the left and right upper and lower extremities and the trunk, and an assessment of deep tendon reflexes (biceps, triceps, quadriceps knee jerk, and ankle jerk) were performed. In patients who reported urinary or stool incontinency, perianal, and perigenital sensory exams were performed with a digital rectal examination, as well as an assessment of residual urine with ultrasound or catheterization. The neurological outcome was measured in means of improvement, and unchanged or worsened neurological status following surgery, as compared to recorded neurological deficits prior to surgery. 

Pain assessment was performed using Visual Analogue Score scale (VAS scale). VAS was routinely obtained prior to surgery and at discharge from the hospital. All statistical computations were performed using SPSS Statistics 23 (IBM, Hamburg, Germany). In descriptive statistics, for parametric variables, such as gender, infection parameters, including the WBC count of CRP, hospital stay or duration of antibiotic therapy, minimal and maximal value with a mean and standard deviation (STD) were calculated. For non-parametric variables (favorable or non-favorable outcomes and the influence of the presence or absence of empyema in the outcome), calculation of frequencies in distinct classes and their percentage were defined, as well as the cross-product and Pearson’s Chi-Squared test and Fisher’s Exact test for defining the significance of the differences in the frequencies in the classes or groups (for example, determination of significance of differences between patients with improved, unchanged or worsened neurological statuses following surgery or the determination of a significance between patients with favorable and non-favorable outcomes, as well as the influence of certain parameters on outcomes, such as smoking, diabetes mellitus and duration of antibiotic therapy). A t-test was used to measure statistically significant differences between the means. To calculate the differences between standard deviations, Leven’s Test for the equality of variances was performed before the t-test. If there was a statistically significant difference between the SDs, the t-test was not performed. An independent sample t-test was used to compare the different mean values between the two groups (favorable and non-favorable outcomes) and paired sample t-tests to compare the variables of two dependent samples for the same patients in different settings (e.g., comparison of parameters before and after surgery), which was used to determine the statistical significance. A correlation analysis was used to determine the correlation variables in the form of a parameter with strength correlation measured as a coefficient between 0 and 1. If the correlation coefficient is higher, i.e., more near to 1, correlation of variables is stronger. Regression analysis showed a quantitative correlation and provided the possibility of prediction of a value of the dependent variable from one or more independent variables. An ANOVA test was used where several variables in the parametric form were tested to determine the difference in significances.

## 3. Results

### 3.1. General Characteristics of the Patients

The general characteristics of the patients are summarized in Table 1. Spondylodiscitis of the cervical spine was defined as an infection of one or more segments from C1/2 to C7/T1; spondylodiscitis of thoracic spine was defined as an infection in the segments T1/T2 to T12/L1, and spondylodiscitis of the lumbosacral spine was defined as an infection in segments from L1/2 to S4 with an infection of psoas muscle. Twenty-six patients had an infection of more than one segment of the spine. These patients were classified as cervical, lumbar, or thoracic according to the predominant site of pathology. Single level spondylodiscitis was defined as an infection of one intervertebral disc.

### 3.2. Symptoms and Neurological Status

Symptoms and neurological statuses are summarized in Table 2. Pain was the most common symptom, present in 225 (94.9%) of patients. One hundred and seventy-two patients, or 72.6%, had neurological deficits. Time from onset of symptoms to diagnosis ranged from one to 67 days, with mean value of 17.9 days (SD: 14.8). Patients with epidural abscess had significantly higher rates of neurological deficits than the patients without abscess (*p* < 0.05). 

### 3.3. Laboratory Findings and Microbiology

All patients had increased values of laboratory infection parameters in the blood analysis. Forty-six patients, or 19.4%, had sepsis with multiple organ failures. In 180 patients (76%), microorganisms were isolated from the intraoperative specimen. The most common pathogen was *Staphylococcus aureus* (77 patients, or 32.5%; of which 13 patients, or 5.5%, were diagnosed with *Methicillin-resistant Staphylococcus aureus* (MRSA). Positive blood culture with an isolation of the pathogen was found in 55 patients (23.2%), and the most common pathogen was *Staphylococcus aureus* (26 patients). There were no cases of multiple pathogens. In 57 patients (24%), the pathogen could not have been isolated from the intraoperative specimen. The diagnosis of the infectious disease in these cases was set in accordance with clinical findings, elevated infection parameters in the blood analysis, neuroradiological diagnosis of spondylodiscitis and neuropathological findings from the intraoperative specimen confirming an inflammation reaction in the tissue. The laboratory and microbiological findings are summarized in Table 3. 

### 3.4. Operative Therapy

Details of the operative treatment are summarized in Table 4. 

**Figure 1:**
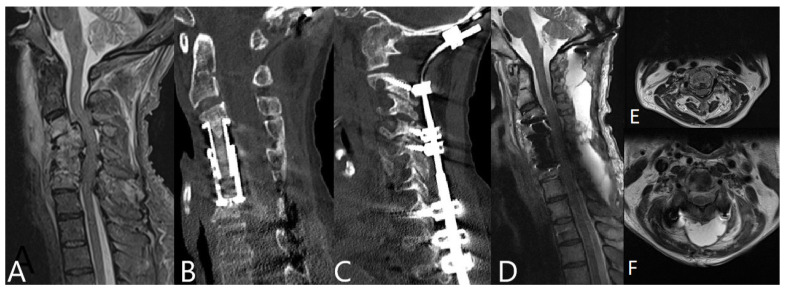
50-year-old male patient with spondylodiscitis due to an infection with methicillin-resistant *Staphylococcus aureus* (MRSA) of C3/4, C4/5, and C5/6, intraspinal and prevertebral empyema, and compression of the spinal cord. Corpectomy of C3, C4, C5, and C6 was performed with implantation of the expandable cage following posterior cervical and thoracic stabilization with lateral mass and pedicle screws with an occiput plate. (**A**). preoperative T2 weighted MRI of the cervical spine. (**B**,**C**). postoperative sagittal CT of the cervical spine shows the spinal construct. (**D**). postoperative T2 weighted MRI of the cervical spine shows restored alignment without signs of infection 3 months following surgery. (**E**). T2 weighted MRI of the cervical spine, axial view at C3 level preoperative and (**F**). postoperative, showing sufficient decompression following ventral decompression and stabilization.

**Figure 2:**
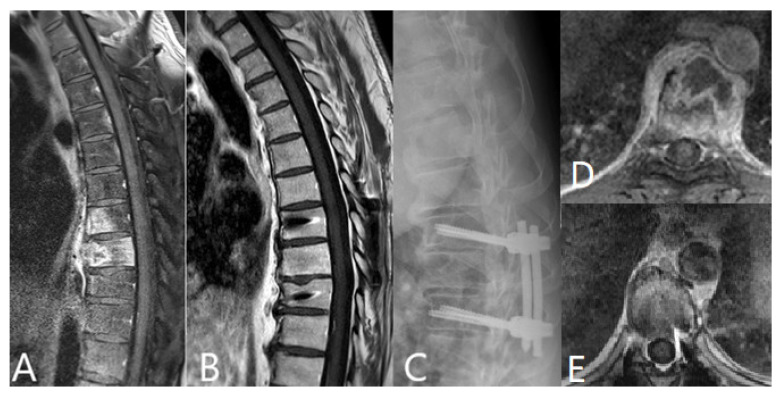
44-year-old male patient with spondylodiscitis of the thoracic spine due to an infection with *Mycobacterium tuberculosis*. (**A**). preoperative T1 post-contrast MRI of the thoracic spine shows spondylodiscitis in Th 7/8 and Th 8/9. (**B**). postoperative T1 post-contrast of the thoracic spine shows the complete resolution of the infection 6 months following surgery. (**C**). postoperative X-ray of the thoracic spine shows the spinal construct with screws and rods in T7-T9. (**D**). Axial T1-weighted post-contrast MRI of the spine at Th7 level shows infection predominantly in ventral portion of the vertebral body. (**E**). T1-weighted post-contrast MRI of the spine at Th7 level 6 months following surgery shows complete resolution of the infection.

**Figure 3:**
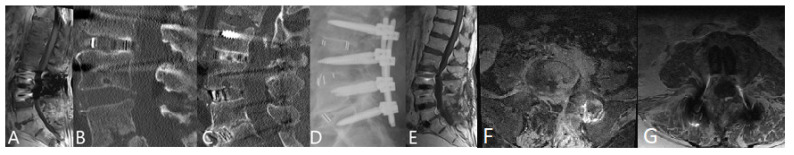
66-year-old male patient with spondylodiscitis and epidural empyema due to an infection with pseudomonas aeruginosa at L4/5 and L5/S1 following the stabilization of L3/4 with an intervertebral cage due to degenerative spinal canal stenosis. (**A**). preoperative T1 post-contrast MRI of the lumbar spine shows spondylodiscitis at L4/5 and L5/S1 with intraspinal empyema. (**B**). preoperative CT of the lumbar spine shows infectious degeneration of L4/5 segment. (**C**). postoperative lumbar spine following extension of the fusion to S1 with cage implantation in L4/5 and L5/S1. (**D**). postoperative sagittal X ray of the lumbar spine shows the spinal construct. (**E**). postoperative MRI of the lumbar spine shows the complete resolution of infection 9 months following surgery. (**F**). T1 weighted post-contrast of the MRI of the lumbar spine at L5 level shows intraspinal empyema. (**G**). Postoperative T2 weighted MRI of the spine shows infection resolution.

All patients with spondylodiscitis of the cervical spine received instrumentation, and of these, 23 patients received dorsoventral stabilization (360° fusion). In patients with spondylodiscitis of the thoracic and lumbar spine, decompression surgery with empyema evacuation was initially performed in 54 patients (in 26 patients as immediate surgery in less than 12 h following admission and in a further 28 patients following admission in 12–24 h). One hundred and thirty-eight patients in thoracic and lumbar spine groups received dorsal instrumentation. Illustrative cases are demonstrated in Figure 1, Figure 2 and Figure 3.

Surgical complications occurred in 51 patients (21.5%). Twenty-eight patients (11.8%) underwent revision surgery due to hardware failure (screw correction and dislocation of the implant). In 44 cases, wound healing deficits occurred (18.6%), 5 patients had cerebrospinal fluid leaks (2.1%), and 8 patients had postoperative hematoma (3.4%). Eighteen patients (7.6%) required three or more surgeries due to complications. 

Recurrent spondylodiscitis occurred in 18 patients (7.6%), and of these, 9 cases had treated and adjacent segments and another 9 cases had adjacent segments only. Adjacent segment disease, which required further stabilization surgery, occurred in 11 patients during the follow-up (4.7%), and 7 patients were treated conservatively for recurrent spondylodiscitis. 

### 3.5. Etiology and Concomitant Infections

Sixty-two (26.2%) patients had postoperative spondyodiscitis following surgery on the spine to address degenerative disease with localization of the infection on the operated segment of the spine. All of these patients received a nucleotomy in the initial surgery. From this number, 18 patients underwent prior surgery at our department and 44 at other institutions. In 175 (73.8%) patients, there were no previous surgeries on the spine. 

One hundred and forty-eight patients (62.4%) had isolated spondylodiscitis, and from this number, 45 patients had spondylodiscitis following spine surgery, and 89 patients, or 37.6%, had concomitant infections other than infection of the psoas muscle (*n* = 35), which were counted as infectious diseases of the lumbar spine, together with spondylodiscitis. Concomitant infections are summarized in Table 5.

One hundred seventy or 71.7% of patients who did not develop adequate remission of infection following initial operative and antibiotic therapy, or where the pathogen could not have been isolated, were fully screened for further infection foci. In 81 patients (34.2%), screening was negative, in 89 patients, one or more concomitant infection sites were found. Fifteen patients had one or more infection foci in the oral cavity, 8 patients in the nasal cavity, 10 patients with endocarditis, 40 patients had urinary tract infection, 48 patients had pneumonia, and 8 patients had other infections.

In 10 patients, endocarditis was diagnosed with trans-esophageal echocardiography. Eight patients with endocarditis were treated with IV antibiotics only and an additional two patients underwent surgery for valve reconstruction with antibiotic therapy. 

### 3.6. Antibiotic Therapy

Broad spectrum antibiotic therapy was applied in all patients following surgery with the standard use of Vancomycin 3 × 1 g IV or Linezolid 2 × 600 mg IV, +Meropenem 3 × 1 g i.v + Fosfomycin 3 × 5 g IV Specific antibiotic therapy directed to the pathogen could then be applied in 180 patients (76%) after isolation of the microorganisms from the intraoperative specimen. In all other cases, broad spectrum IV antibiotic therapy was applied continuously until the antibiotic therapy could be switched to oral application. 

Ninety-eight patients, or 41.4%, received antibiotic therapy at other hospitals prior to the diagnosis of spondylodiscitis due to the symptoms of the infection. From this number, in 57 patient microorganisms could not have been isolated from the intraoperative specimen or blood culture. Details of the duration of antibiotic therapy are summarized in Table 6. Ninety-one patients received cumulative antibiotic therapy for a period longer than 6 weeks (42 days).

### 3.7. Outcome

#### 3.7.1. Healing of Infection and Recovery of Infection Parameters

**Figure 4:**
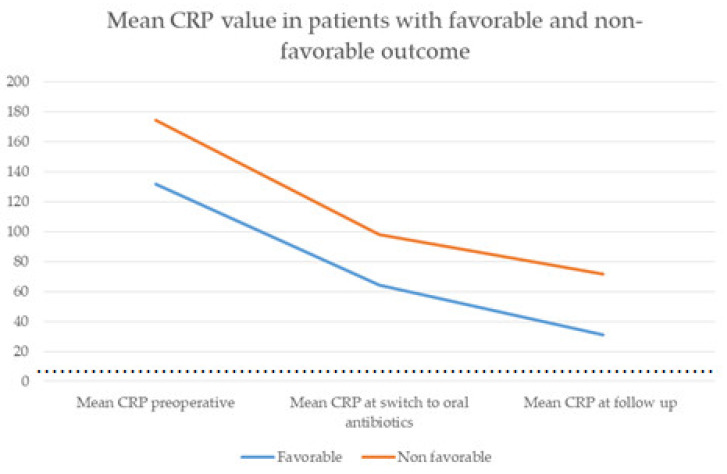
Mean CRP value in patients with favorable and non-favorable outcome. Graph shows course of CRP-value in patients with favorable (blue line) and no- favorable outcome (orange line). Threshold of CRP value of 5 mg/L, which is considered to be physiologic, is presented with dotted line. Patients with unfavorable outcome had higher initial CRP values and higher values of CRP at the time of switch to oral antibiotics, compared to the patients with favorable outcome.

Complete healing was defined as normalization of the infection parameters (normalization of leukocyte count and significant fall of C-reactive protein) was achieved in 211 (89%) of cases. The mean postoperative leucocyte count at follow-up was 8.03 G/L (SD: 4.05) and was statistically significant compared to the preoperative value of 11.66 (SD: 5.27) (match paired t test, t = 11.071; *p* < 0.001). The mean time of normalization of the leukocyte count (<10 G/L) was 9.5 days (SD: 44.56). Mean postoperative CRP was 45.4 mg/L (SD: 68.85) and was statistically significantly reduced in comparison to the preoperative value of 160.8 (SD: 159.52) (t = 15.107; *p* < 0.001). In 96 patients, CRP was normal (<5 mg/L) at the follow-up (40.5%). The mean CRP value at the point of switch of antibiotic therapy from IV to oral was 76.5 mg/L (SD ± 48.35) for all patients. The mean CRP value at the start of oral antibiotic therapy in patients with favorable outcomes was 64.3 mg/L, as compared to 98 mg/L in patients with unfavorable outcomes without statistical significance. Antibiotic therapy was switched to oral use when the initial CRP fell to an average of 53.1% of the initial value (54.5% for patients with favorable and 52% for patients with unfavorable outcomes). Mean postoperative CRP for patients with unfavorable outcomes was 71.8 mg/L/SD: 96.71) and mean postoperative CRP for patients with favorable outcomes was 31.2 (SD: 42.81) (Figure 4). Thirteen patients, or 5.5%, had infections with methicillin-resistant *Staphylococcus aureus* (MRSA), 10 of them had unfavorable outcomes, with 5 dying during their primary hospital stay. All 21 patients who died during the hospital stay had sepsis with multiple organ failures and two or more concomitant infections. 

#### 3.7.2. Pain Reduction and Neurological Status

Postoperative pain reduction was significant, with a mean postoperative VAS of 2.03 (SD: 0.19) (Pearson Chi Square χ^2^ = 0.262, *p* < 0.001, ANOVA Regression Coefficient R = 0.778). Immediately following surgery, the neurological status improved in 38 of 211 patients, in 160 it remained unchanged, and in 13 it worsened. At follow-up, the neurological status improved in 101 patients (42.6%), remained unchanged in 95 patients (40%) and worsened in 15 patients (6.3%). One hundred and fifty-six patients (65.8%) were able to walk at the last follow-up. The number of patients with an improved neurological status was significant (χ^2^ = 9.981, *p* = 0.002).

#### 3.7.3. Outcome Measurement

Significant outcome parameters are summarized in Table 7. A favorable outcome was defined as a complete regression of infection (significant decline of C-reactive protein), significant reduction of pain symptoms (VAS Score), improved neurological status or unchanged neurological status in patients without deficits prior to surgery, with ability to walk. One hundred and fifty-six patients (65.8%) had favorable outcomes. Favorable outcomes were shown in patients without concomitant infections (*n* = 148, χ^2^ = 7.948, *p* = 0.005), with a completely normalized CRP value (*n* = 96, χ^2^ = 5.410; *p* = 0.02) and in patients who received antibiotic therapy over a period of more than six weeks (*n* = 91 or 38.4%, Corr = −0.159, χ^2^ = 5.733 *p* = 0.017). The number of patients with favorable outcomes vs. number of patients with unfavorable outcomes was significant (χ^2^ = 18.941, *p* < 0.01).

An unfavorable outcome was defined as an incomplete regression of infection (decline of C-reactive protein concentration not significant) with or without recurrent spondylodiscitis and/or unchanged or worsened neurological status without the ability to walk. Eighty-one patients (34.2%) had an unfavorable outcome, including 26 patients who died (21 in the initial hospital stay and 5 up to two years following surgery) and 15 patients with worsened neurological outcomes. Unfavorable outcomes were shown in patients with higher preoperative CRP values (mean CRP of 174.35 mg/L vs. 131. 84 mg/L compared to patients with favorable outcome), with postoperative spondylodiscitis (corr = −0.155; χ^2^ = 5.724, *p* < 0.02) and with recurrent spondylodiscitis (Corr = −0.184, χ^2^ = 0.004, *p* < 0.01).

The preoperative leucocyte count, age and gender, microbiological diagnosis (isolation of pathogen in operative specimen or blood culture), presence of empyema and risk factors such as diabetes mellitus or smoking, presence of neurological deficits, as well as operative approach (ventral vs. dorsal) did not show a correlation with the outcome (*p* < 0.05). Patients with unfavorable outcomes had more frequent postoperative complications, as compared to patients with favorable outcomes, but this was not statistically significant (*p* < 0.05) (Table 7).

## 4. Discussion

### 4.1. Conservative vs. Operative Treatment of Spondylodiscitis

Despite its retrospective nature, this study allows for several substantial conclusions regarding the neurosurgical management of spondylodiscitis to be drawn. Reported failure rates following conservative management ranged from 12% to 18% [20]. While the infection rate controls are shown to be similar, patients treated early by surgery and antibiotics were hospitalized for fewer days and required less antibiotics than those treated with antibiotics alone [21]. Osteosynthesis should be preferred for spondylodiscitis with osteolysis and spinal instability because it allows for early mobilization and rehabilitation, prevents spinal deformity and does not hamper the regression of infections [12]. Our cohort consisted of patients with whom conservative treatment was discussed as an alternative option to surgery, but was not applied for the reasons mentioned above. Mortality and morbidity were similar for patients with conservative and operative treatment [22]. In the case of infection with multiresistant bacteria, mortality rates were reported to be up to 38% for conservative treatment [23,24]. In our cohort, 13 patients, or 5.5%, had infections with MRSA, 10 of them had unfavorable outcomes with five who died during their primary hospital stay. In our department, conservative therapy of spondylodiscitis was always discussed with patients as a therapy alternative. In our retrospective review, we excluded very few cases (<10) in the study period, which were treated conservatively since the comparison with a far larger number of operatively treated patients would be not practical. These were all patients of advanced age (over 85 years old) with several concomitant diseases and who were in a reduced general condition. The recently developed Brighton Spondylodiscitis Score (BSDS) helps to identify patients with spondylodiscitis who would fail non-operative management and would benefit from early surgery [25]. Immune-compromised patients with comorbidities and concomitant diseases, such as endocarditis as well as signs of vertebral collapse and the presence of motoric neurological deficits, have been identified as high risk patients that require surgical interventions [25]. BSDS was developed in the United Kingdom in a healthcare system that is different to the one in Germany. A recent retrospective study on 91 patients with spondylodiscitis from New Zealand failed to externally validate the BSDS [26]. Caution was advised when adopting treatment algorithms developed in other healthcare systems that may comprise significantly different patient and pathogen characteristics [26]. While recent study has demonstrated that early surgery with antibiotics for patients with pyogenic spondylodiscitis has been shown to shorten hospitalization and requires less antibiotics than those treated with antibiotics alone, with better functional outcomes [21], one important drawback of the surgical treatment of the therapy of spondylodiscitis, as compared to the conservative treatment, has not been discussed—i.e., the question of the cost of surgical therapy (especially in the case of multiple surgeries) and the cost–benefit compared to conservative treatment alone. Some authors advocate intractable pain as an indication for surgical therapy, regardless of the presence of instability, empyema, and neurological deficits [2,27,28,29]. 

### 4.2. General Characteristics, Neurological Deficits and Mortality

Age and gender distributions are similar those in previous studies [1,16,19,21,30,31,32,33]. The lumbosacral spine is the most common site of infection [1,19,20,30,34]. Mortality is high, at 8.9%, and has been described as ranging from 1.8% to 15% [1,19,32,35]. Short-term mortality was shown to be related to empyema and neurologic deficits [5]. The number of patients with neurological deficits ranges from 35% [19,36] to 40% [34]. Severe neurological deficits are more common in the cervical and thoracic spine, in infection with *S. aureus*, in the presence of epidural abscess, and when CRP is higher than 150 mg/L [37]. The presence of epidural empyema shows a correlation with neurological deficits [37]. Discitis and osteomyelitis are underdiagnosed due to the nonspecific initial presentation of back pain [38]. Recent epidemiological studies have shown that the median diagnostic delay in the case of pyogenic spondylodiscitis ranges from 30 days [1] to 69.4 days [34]. The time from the onset of symptoms to the diagnosis of spondylodiscitis was 17.88 days, and even 98 patients, or 41.4%, patients who were referred to our department from other hospitals received antibiotic therapy for unclear infection prior to surgery. One study showed that the time from the onset of symptoms to the first surgical treatment was about 69.4 days, and did not change significantly over the course of the past two decades [34]. This shows that spondylodiscitis remains an underestimated cause of infection disease and it also explains the high number of patients without the isolation of the pathogen due to prior antibiotic therapy. Staphylococcus aureus is the most common pathogen, which is consistent with previous studies [1,19,39]. A high–complete healing rate was also consistent with a recent study, which included 207 patients with spondylodiscitis with a reported healing from infection rate of 90.9% [1].

### 4.3. Value of Measurement of Laboratory Infection Parameters

To date, there are no guidelines or controlled trials that define optimal antibiotic therapy, but there is consensus that antibiotic therapy should be discontinued when laboratory infection parameters (WBC count, CRP concentration) normalize [19,40,41]. In our experience, discontinuation prior to the complete normalization of the infection parameters is primarily conducted in rehabilitation centers when clinical improvement occurs. The mean preoperative CRP value is in our study was higher than in previous studies [19,42] and it has shown a clear correlation with unfavorable outcomes. Higher initial CRP showed a correlation with the relapse of spondylodiscitis in previous studies [43]. Patients with a normalized CRP value had a clear correlation with favorable outcomes in our series. We did not routinely measure procalcitonin or infection estimation and response control, since it has been shown that it is not useful as a diagnostic tool or monitoring parameter for spondylodiscitis, and is not useful for the discrimination between a bacterial infection and aseptic inflammation of the spine [44]. Higher initial CRP showed a correlation with recurrence [43]. Intravenous antibiotic therapy was applied in a range of 10 days to 6 weeks [19,41] with oral antibiotics taken from 6 weeks to three months [1,16,40,41]. Antibiotic therapy for a period of longer than six weeks had favorable outcomes, which is in accordance with previous studies [45]. The overall relapse rate was reported to be up to 6.6%; notably, this rate was higher in patients who received antibiotics for ≤6 weeks [46].

One recent study found that the identification of the pathogen could only have been conducted in 22.4% of the patients with pyogenic spondylodiscitis [47]. Postoperative antibiotic therapy was managed against the result of the susceptibility test, or was empirically given to patients with negative cultures with all antibiotic therapy IV initiated for 4–6 weeks and orally for 6 weeks [47].

### 4.4. Surgical Therapy

A surgical approach needs to be tailored on an individual basis [19,40]. We favored early surgical therapy, i.e., within 24 h of admission. Evacuation of epidural empyema within 24 h appears to have had a relative advantage over delayed surgery with regard to the discharge of the neurological grade [48]. Anterior cervical surgery with debridement and antibiotics achieved complete healing, while the use of PEEK cages had no negative effects [32,49]. Additional dorsal stabilization is recommended in cases of spinal instability and after corpectomy [32]. Pedicle screw fixation with interbody fusion is an effective technique for patients with spondylodiscitis [50].

The complication rate in our study was 21.5%. Revision surgeries of the posterior spinal constructs have been described in the literature as being up to 22% [31]. Other studies have shown lower complication rates [19,33,51,52]. This could be explained by a high percentage of patients who have concomitant diseases and thus underwent two-staged surgery, which in turn increased the risk of wound healing deficits. One of the measures that could have been undertaken to prevent this is the reduction of two-stage surgery in a way where early surgery includes an immediate stabilization when deemed to be necessary. One further measure that is currently in use in our department is the use of vancomycin powder, since pooled clinical data supports this to prevent surgical site infections in adult spine surgeries without safety concerns [53].

### 4.5. Influence of Concomitant Infections

The presence of concomitant infections like pneumonia, urinary tract infection, and endocarditis was related to unfavorable outcomes. This justifies the establishment of a protocol to search for a further infection focus. We have established a standard using trans-esophageal echocardiography (TEE), contrast-enhanced CT for the thorax and abdomen, as well as clinical examination of the nasal and oral cavity in search of an infection focus. The use of TEE in screening for endocarditis in the treatment of spondylodiscitis has been recommended [18], as the mortality rate in patients with infective endocarditis was significantly higher [1]. Oral cavity infections have been identified as a frequent source of spondylodiscitis, with smoking as the most relevant associated risk factor [54]. Detailed diagnostic work-up, including a mandatory maxillofacial assessment, is strongly recommended [54]. CRP has been shown to be a predictable serum parameter in patients with spondylodiscitis, while the WBC count is unspecific [55]. One recent study proposed that, if CRP does not fall after the third postoperative day, antibiotic treatment should be reassessed, and concomitant infections screened [55]. Implementation of a weekly infection conference consisting of a surgeon, medical microbiologist, infectious disease specialist and pathologist has shown the improvement of treatment of spondylodiscitis with reduced days of total antibiotic treatment and more frequent open transpedicular screw placement with less involved spinal segments [56].

### 4.6. Outcome Parameters

Patients with primary, noncomplicated spondylodiscitis have the highest likelihood of absolute recovery [30]. Microbiological diagnosis has been postulated to be the main predictive factor for successful treatment [1,16]. Identification of the pathogen in the intraoperative specimen or blood culture showed no correlation with favorable outcomes in our study. *Staphylococcus aureus* was the most common pathogen, which is similar to previous studies [39]. Complete healing in previous studies was achieved in up to 77.6% of patients [1]. Incomplete healing poses the risk of recurrence. Neurologic impairment at diagnosis [1] has been identified as a poor prognostic factor for spondylodiscitis, which could not be shown in our results. Rather, our results suggest that patients with spondylodiscitis following spine surgery are at risk of unfavorable outcomes, as compared to patients with primary spondylodiscitis, which is contrary to a recent study [57]; however, prospective clinical trials will be mandatory in order to better understand the pathogenesis and to develop evidence-based treatment recommendations. A previous study has shown that pyogenic vertebral osteomyelitis (PVO) with no identified microorganisms has less systemic inflammatory responses than microbiologically confirmed osteomyelitis with a lower treatment failure rate [46]. In cases of culture-negative spondylodiscitis, prolonged antibiotic therapy for at least 8 weeks has been proposed [46]; however, one further study has shown that culture-negative cases required less parenteral antibiotics [58].

### 4.7. Limitations of the Study 

The limitations of this study are its relatively small number of patients and its retrospective character; however, future prospective studies are needed to accurately evaluate the prognostic factors that are optimal for the surgical treatment of spondylodiscitis. A further limitation is the lack of a control group of patients with conservative treatment; however, the current clinical treatment of infectious diseases of the spine shifted to the inclusion of surgical therapy as this enables early mobilization. 

## 5. Conclusions

Our conclusion supports the current guideline facts. Spondylodiscitis should be included in the differential diagnosis of every unclear infectious disease associated with neck or back pain, especially in elderly multimorbid patients. Indications for surgical therapy should be broad due to the possibility of surgically removing the infection focus, microbiological diagnosis of the intraoperative specimen, as well as the possibility of early mobilization of the patient. Complete regression of infection is essential for successful treatment. Effective infection regression could be achieved with surgical therapy through instrumentation in selected cases following the application of antibiotic therapy for a minimum of six weeks, or even better, until complete normalization of the infection parameters. The absence of concomitant infections shows a good correlation with favorable outcomes, and so we strongly recommend regular screening for all patients. Our experience has shown that screening for concomitant infections should include trans-esophageal echocardiography, oral and nasal cavity examination, urinary status and a post-contrast CT of the thorax and abdomen to identify further infection foci.

Patients with significant pain reduction, improved neurological status, or unchanged neurological status in the case of patients without deficits prior to surgery, with ability to walk, without concomitant infections, and with completely normalized CRP values who have received antibiotic therapy for a period of more than six weeks show favorable outcomes. Unfavorable outcomes were shown in patients with higher preoperative CRP values in patients with spondylodiscitis following spinal surgery and in patients with recurrent spondylodiscitis. 

## Figures and Tables

**Table 1:** General characteristics of the patients.

Patient Characteristics	Number
Overall number of patients (*n*)	*n* = 237 (100%)
Gender	
Male	150 (63.3%)
Mean age (years)	71.4 (Standard deviation SD ± 12.9)
Mean follow-up (months)	31.62 (SD ± 19.5)
MRI of the spine at follow-up	125 patients (52.7%)
Average duration of hospital stay (days)	14.1 (SD ± 8.2)
Mortality	
Death during the initial hospital stay	21 patients (8.9%)
Death up to two years following discharge	5 (2.1%)
Spine level (*n*)	
Cervical	45 patients (19%)
Thoracic	73 (30.8%)
Lumbosacral	119 (50.2%)
Psoas muscle abscess	35 (14.8%)
Spinal levels (*n*)	
One level	174 patients (73.4%)
Two levels	44 (18.7%)
Three levels	7 (3%)
Four or more levels	12 (5%)
Mean number of infected levels (*n*)	1.33 (SD: 1.204)
Epidural empyema (*n*)	
Present	146 (61.6%)
Absent	91 (38.4%)
Mean number of segments of empyema spread (*n*)	1.43 (SD 2.165)
Concomitant diseases	185 patients (78%)
Two or more diseases	155 (65.4%)
Arterial hypertension	145 (61.2%)
Renal insufficiency	70 (29.5%)
Lung diseases	81 (34.2%)
Diabetes mellitus	74 (31.2%)
Malignant primary tumors	46 (19.4%)
Obesity	40 (16.9%)
History of alcohol abuse	18 (7.6%)
Smokers	26 (11%)
History of drug abuse	9 (3.8%)

**Table 2:** Symptoms and neurological statuses.

Patient Characteristics	Number
Pain preoperative	225 patients (94.9%)
Mean preoperative VAS	8.17 (SD: 2.5).
Mean postoperative VAS	2.03 (SD: 0.19), *p* < 0.001
Neurological deficits preoperative	172 patients (72.6%)
Motor deficits with paresis of one or more muscles	63 patients (26.6%)
Motor deficits with paraparesis	22 patients (9.3%)
Motor deficits with tetraparesis	4 patients (1.7%)
Sensory deficits	75 patients (31.6%)
Ataxia	30 patients (12.7%)
Time from onset of symptoms to diagnosis	17.9 days (range 1–67 days, SD: 14.8).
Neurological status at follow-up	
Improved	101 patients (42.6%), *p* = 0.002
Unchanged	95 patients (40%)
Worsened	15 patients (6.3%)

**Table 3:** Laboratory and microbiological findings.

Laboratory Findings	Value
Mean leukocyte (WBC) count preoperative (G/L)	11.66 (SD: 5.28)
Mean leukocyte (WBC) count postoperative (G/L)	8. 03 (SD: 4.05), *p* < 0.001
Mean C-reactive protein (CRP) preoperative (mg/L)	160.8 (SD: 159.52)
Mean C-reactive protein (CRP) postoperative (mg/L)	45.44 (SD: 68.85), *p* < 0.001
Microbiological findings	
Isolation of pathogen in intraoperative specimen (*n* = 237) No	57 patients (24%)
Yes	180 patients (76%)
*Staphylococcus aureus*	77
*MRSA*	13
*Streptococcus spp.*	8
*Enterococcus spp.*	8
Other	74
Isolation of pathogen in blood culture (*n* = 237)	
No	182 patients (76.8%)
Yes	55 patients (23.2%)
*Staphylococcus aureus*	26 (11%)
Pathogens in patients who died during the initial hospital stay	21 patients
*Staphylococcus aureus*	5
*MRSA*	2
*Streptococcus spp.*	1
No pathogen isolation	13

**Table 4:** Operative treatment.

Operative Therapy	Number of Patients
Overall number of patients	237 (100%)
Cervical spine	45
Ventral discectomy with PEEK (Polyetheretherketon) cage	31
Corpectomy with Titanium expandable cage	14
Additional dorsal stabilization	23
Thoracic and lumbar spine	
Decompression and empyema evacuation without stabilization	54
Dorsal stabilization	138
Without cage	36
With cage	102
TLIF (Transforaminal interbody fusion) PEEK Cage	45
TLIF Titan Cage	35
XLIF (Extreme lateral interbody fusion) PEEK Cage	22
Dorsal stabilization overall	161
One segment	25
Two segments	47
Three and more segments	89
Single surgery	122 (51.5%)
Multiple surgeries	115 (48.5%)
Early surgery (within 24 h)	222 (93.7%)
Psoas muscle abscess	35
CT-guided punction	19
Conservative therapy	16
Surgical complications	51 (21.5%)
Hardware failure	28
Wound healing deficits	44

**Table 5:** Concomitant infections.

Concomitant Infections	Patients
Overall number of patients	237 (100%)
Present	89 (37.6%)
Pneumonia	48 (20.25%)
Urinary tract infections	40 (16.9%)
Endocarditis	10 (4.2%)
*Stap aureus*	7
*MRSA*	1
*Haemophilus influenzae*	1
*Pseudomonos aeruginosa*	1
Sepsis	46 (19.4%)

**Table 6:** Antibiotic therapy.

Antibiotic Therapy	Number (Days)
Mean time of application of empiric IV antibiotic therapy	9.6 ± 3.4 (SD:11)
Mean total time of application of IV antibiotic therapy (empiric and tailored)	20.5 (SD: 22, range 2–297 days)
Oral antibiotic therapy in cases where pathogens could have been isolated from the intraoperative specimen	47.4 ± 0.9 (SD:45.2).
Cummulative oral antibiotic therapy in all cases	58.9 (SD: 46.3, range 2–462 days)
Mean time of cummulative application of antibiotic therapy	73.9 (SD: 54)

**Table 7:** Outcome parameters.

Outcome Parameters	Outcome (*n* = 237 Patients)	*p*-Value
Significant decline of C-reactive protein, significant decline of VAS score, improved or unchanged neurological status	Favorable (*n* = 156)	*p* = 0.005
Absence of concomitant infections	Favorable (*n* = 148)	*p* = 0.005
Completely normalized CRP_value (CRP less than 5 mg/L)	Favorable (*n* = 96)	*p* = 0.02
Antibiotic therapy longer than 6 weeks	Favorable (*n* = 91)	*p* = 0.017
Higher preoperative CRP value	Unfavorable (*n* = 85)	*p* = 0.009
Postoperative spondylodiscitis	Unfavorable (*n* = 62)	*p* < 0.02
Recurrent spondylodiscitis	Unfavorable (*n* = 18)	*p* < 0.01

## Data Availability

Portion of the results presented in this manuscript have been presented as an oral presentation entitled “Neurosurgical Management and Outcome Parameters in 237 Patients with Spondylodiscitis” on the Annual Meeting of German Society of Neurosurgery (71. Jahrestagung der Deutschen Gesellschaft für Neurochirurgie (DGNC) DGNC Online, Lübeck, June 2020). Abstract can be found online (7 June 2021): https://www.egms.de/static/de/meetings/dgnc2020/20dgnc081.shtml.
